# Male breast cancer in *BRCA1* and *BRCA2* mutation carriers: pathology data from the Consortium of Investigators of Modifiers of *BRCA1/2*

**DOI:** 10.1186/s13058-016-0671-y

**Published:** 2016-02-09

**Authors:** Valentina Silvestri, Daniel Barrowdale, Anna Marie Mulligan, Susan L. Neuhausen, Stephen Fox, Beth Y. Karlan, Gillian Mitchell, Paul James, Darcy L. Thull, Kristin K. Zorn, Natalie J. Carter, Katherine L. Nathanson, Susan M. Domchek, Timothy R. Rebbeck, Susan J. Ramus, Robert L. Nussbaum, Olufunmilayo I. Olopade, Johanna Rantala, Sook-Yee Yoon, Maria A. Caligo, Laura Spugnesi, Anders Bojesen, Inge Sokilde Pedersen, Mads Thomassen, Uffe Birk Jensen, Amanda Ewart Toland, Leigha Senter, Irene L. Andrulis, Gord Glendon, Peter J. Hulick, Evgeny N. Imyanitov, Mark H. Greene, Phuong L. Mai, Christian F. Singer, Christine Rappaport-Fuerhauser, Gero Kramer, Joseph Vijai, Kenneth Offit, Mark Robson, Anne Lincoln, Lauren Jacobs, Eva Machackova, Lenka Foretova, Marie Navratilova, Petra Vasickova, Fergus J. Couch, Emily Hallberg, Kathryn J. Ruddy, Priyanka Sharma, Sung-Won Kim, kConFab Investigators, Manuel R. Teixeira, Pedro Pinto, Marco Montagna, Laura Matricardi, Adalgeir Arason, Oskar Th Johannsson, Rosa B. Barkardottir, Anna Jakubowska, Jan Lubinski, Angel Izquierdo, Miguel Angel Pujana, Judith Balmaña, Orland Diez, Gabriella Ivady, Janos Papp, Edith Olah, Ava Kwong, Hereditary Breast and Ovarian Cancer Research Group Netherlands (HEBON), Heli Nevanlinna, Kristiina Aittomäki, Pedro Perez Segura, Trinidad Caldes, Tom Van Maerken, Bruce Poppe, Kathleen B. M. Claes, Claudine Isaacs, Camille Elan, Christine Lasset, Dominique Stoppa-Lyonnet, Laure Barjhoux, Muriel Belotti, Alfons Meindl, Andrea Gehrig, Christian Sutter, Christoph Engel, Dieter Niederacher, Doris Steinemann, Eric Hahnen, Karin Kast, Norbert Arnold, Raymonda Varon-Mateeva, Dorothea Wand, Andrew K. Godwin, D. Gareth Evans, Debra Frost, Jo Perkins, Julian Adlard, Louise Izatt, Radka Platte, Ros Eeles, Steve Ellis, EMBRACE, Ute Hamann, Judy Garber, Florentia Fostira, George Fountzilas, Barbara Pasini, Giuseppe Giannini, Piera Rizzolo, Antonio Russo, Laura Cortesi, Laura Papi, Liliana Varesco, Domenico Palli, Ines Zanna, Antonella Savarese, Paolo Radice, Siranoush Manoukian, Bernard Peissel, Monica Barile, Bernardo Bonanni, Alessandra Viel, Valeria Pensotti, Stefania Tommasi, Paolo Peterlongo, Jeffrey N. Weitzel, Ana Osorio, Javier Benitez, Lesley McGuffog, Sue Healey, Anne-Marie Gerdes, Bent Ejlertsen, Thomas V. O. Hansen, Linda Steele, Yuan Chun Ding, Nadine Tung, Ramunas Janavicius, David E. Goldgar, Saundra S. Buys, Mary B. Daly, Anita Bane, Mary Beth Terry, Esther M. John, Melissa Southey, Douglas F. Easton, Georgia Chenevix-Trench, Antonis C. Antoniou, Laura Ottini

**Affiliations:** Department of Molecular Medicine, Sapienza University of Rome, Viale Regina Elena, 324, 00161 Rome, Italy; Centre for Cancer Genetic Epidemiology, Department of Public Health and Primary Care, School of Clinical Medicine, University of Cambridge, Cambridge, UK; Laboratory Medicine Program, University Health Network, Toronto, ON Canada; Department of Laboratory Medicine and Pathobiology, University of Toronto, Toronto, ON Canada; Department of Population Sciences, Beckman Research Institute of City of Hope, Duarte, CA USA; Peter MacCallum Cancer Institute, East Melbourne, Australia; Women’s Cancer Program at the Samuel Oschin Comprehensive Cancer Institute, Cedars-Sinai Medical Center, Los Angeles, CA USA; Familial Cancer Centre, Peter MacCallum Cancer Centre, Melbourne, Australia; Department of Oncology, The University of Melbourne, Melbourne, VIC Australia; University of Pittsburgh School of Medicine, Pittsburgh, PA USA; UPMC Cancer Center, Pittsburgh, PA USA; Department of Medicine, Abramson Cancer Center, Perelman School of Medicine at The University of Pennsylvania, Philadelphia, PA USA; Department of Epidemiology and Biostatistics, Abramson Cancer Center, Perelman School of Medicine, University of Pennsylvania, Philadelphia, PA USA; Department of Preventive Medicine, Keck School of Medicine, University of Southern California Norris Comprehensive Cancer Center, Los Angeles, CA USA; Department of Medicine and Genetics, University of California, San Francisco, San Francisco, CA USA; Center for Clinical Cancer Genetics and Global Health, University of Chicago Medical Center, Chicago, IL USA; Department of Clinical Genetics, Karolinska University Hospital, Stockholm, Sweden; Cancer Research Initiatives Foundation, Sime Darby Medical Centre, Subang Jaya, Malaysia; University Malaya Cancer Research Institute, Faculty of Medicine, University Malaya Medical Centre, University Malaya, Kuala Lumpur, Malaysia; Section of Genetic Oncology, Department of Laboratory Medicine, University of Pisa and University Hospital of Pisa, Pisa, Italy; Department of Clinical Genetics, Vejle Hospital, Vejle, Denmark; Section of Molecular Diagnostics, Department of Biochemistry, Aalborg University Hospital, Aalborg, Denmark; Department of Clinical Genetics, Odense University Hospital, Odense C, Denmark; Department of Clinical Genetics, Aarhus University Hospital, Aarhus N, Denmark; Department of Molecular Virology, Immunology and Medical Genetics, College of Medicine, The Ohio State University, Columbus, OH USA; Division of Human Genetics, Department of Internal Medicine, The Comprehensive Cancer Center, The Ohio State University, Columbus, OH USA; Lunenfeld-Tanenbaum Research Institute, Mount Sinai Hospital, Toronto, ON Canada; Department of Molecular Genetics, University of Toronto, Toronto, ON Canada; Center for Medical Genetics, North Shore University Health System, Evanston, IL USA; N.N. Petrov Institute of Oncology, St. Petersburg, Russia; Clinical Genetics Branch, Division of Cancer Epidemiology and Genetics, National Cancer Institute, National Institutes of Health, Rockville, MD USA; Department of Obstetrics and Gynecology, Comprehensive Cancer Center, Medical University of Vienna, Vienna, Austria; Department of Urology, Medical University of Vienna, Vienna, Austria; Department of Medicine, Memorial Sloan-Kettering Cancer Center, New York, NY USA; Clinical Genetics Service, Department of Medicine, Memorial Sloan-Kettering Cancer Center, New York, NY USA; Department of Cancer Epidemiology and Genetics, Masaryk Memorial Cancer Institute, Brno, Czech Republic; Masaryk Memorial Cancer Institute and Faculty of Medicine, Masaryk University, Brno, Czech Republic; Department of Laboratory Medicine and Pathology, Mayo Clinic, Rochester, MN USA; Department of Health Sciences Research, Mayo Clinic, Rochester, MN USA; Department of Oncology, Mayo Clinic, Rochester, MN USA; Department of Hematology and Oncology, University of Kansas Medical Center, Kansas City, KS USA; Department of Surgery, Daerim St. Mary’s Hospital, Seoul, Korea; Department of Genetics, Portuguese Institute of Oncology, Porto, Portugal; Biomedical Sciences Institute (ICBAS), University of Porto, Porto, Portugal; Immunology and Molecular Oncology Unit, Veneto Institute of Oncology IOV - IRCCS (Scientific Institute of Hospitalization and Care), Padua, Italy; Department of Pathology, Landspitali University Hospital and Biomedical Centre (BMC), Faculty of Medicine, University of Iceland, Reykjavik, Iceland; Department of Oncology, Landspitali University Hospital and Faculty of Medicine, University of Iceland, Reykjavik, Iceland; Department of Genetics and Pathology, Pomeranian Medical University, Szczecin, Poland; Genetic Counseling Unit, Hereditary Cancer Program, Biomedical Research Institute of Girona (IDIBGI), Catalan Institute of Oncology, Girona, Spain; Breast Cancer and Systems Biology Unit, Bellvitge Biomedical Research Institute (IDIBELL), Catalan Institute of Oncology, Barcelona, Spain; Department of Medical Oncology, Vall d’Hebron University Hospital, Barcelona, Spain; Oncogenetics Group, Vall d’Hebron University Hospital, Vall d’Hebron Institute of Oncology (VHIO) and Universitat Autònoma de Barcelona, Barcelona, Spain; Department of Pathology, National Institute of Oncology, Budapest, Hungary; Department of Molecular Genetics, National Institute of Oncology, Budapest, Hungary; The Hong Kong Hereditary Breast Cancer Family Registry, Cancer Genetics Center, Hong Kong Sanatorium and Hospital, Hong Kong, China; Department of Surgery, The University of Hong Kong, Hong Kong, China; Department of Obstetrics and Gynecology, University of Helsinki and Helsinki University Hospital, Helsinki, Finland; Department of Clinical Genetics, University of Helsinki and Helsinki University Hospital, Helsinki, Finland; Department of Oncology, San Carlos Clinical Hospital Health Research Institute (IdISSC), San Carlos Clinical Hospital, Madrid, Spain; Molecular Oncology Laboratory, San Carlos Clinical Hospital Health Research Institute (IdISSC), San Carlos Clinical Hospital, Madrid, Spain; Center for Medical Genetics, Ghent University, Ghent, Belgium; Lombardi Comprehensive Cancer Center, Georgetown University, Washington, DC, USA; Department of Tumour Biology, Institut Curie, Paris, France; CNRS UMR5558, Université Lyon 1, Lyon, France; Unité de Prévention et d’Epidémiologie Génétique, Centre Léon Bérard, Lyon, France; Université Paris Descartes, Sorbonne Paris Cité, Paris, France; INSERM U1052, CNRS UMR5286, Centre de Recherche en Cancérologie de Lyon, Université Lyon, Lyon, France; Department of Gynaecology and Obstetrics, Technical University of Munich, Munich, Germany; Institute of Human Genetics, University of Wurzburg, Wurzburg, Germany; Institute of Human Genetics, University Hospital Heidelberg, Heidelberg, Germany; Institute for Medical Informatics, Statistics and Epidemiology University of Leipzig, Leipzig, Germany; University of Dusseldorf, Dusseldorf, Germany; Hannover Medical School, Hannover, Germany; Center for Hereditary Breast and Ovarian Cancer, Center for Integrated Oncology (CIO) and Center for Molecular Medicine Cologne (CMMC), Medical Faculty, University of Cologne and University Hospital Cologne, Cologne, Germany; Department of Gynecology and Obstetrics, Technical University of Dresden, Dresden, Germany; Department of Gynaecolgy and Obstetrics, University Hospital of Schleswig-Holstein, Christian-Albrechts-University of Kiel, Kiel, Germany; Institute of Human Genetics, Charité, Berlin, Germany; Institute of Human Genetics, Leipzig, Germany; Department of Pathology and Laboratory Medicine, University of Kansas Medical Center, Kansas City, KS USA; Genetic Medicine, Manchester Academic Health Sciences Centre, Central Manchester University Hospitals NHS Foundation Trust, Manchester, UK; Yorkshire Regional Genetics Service, Leeds, UK; Clinical Genetics, Guy’s and St. Thomas’ NHS Foundation Trust, London, UK; Oncogenetics Team, The Institute of Cancer Research and Royal Marsden NHS Foundation Trust, Sutton, UK; Molecular Genetics of Breast Cancer, German Cancer Research Center (DKFZ), Heidelberg, Germany; Cancer Risk and Prevention Clinic, Dana-Farber Cancer Institute, Boston, MA USA; Molecular Diagnostics Laboratory, Institute of Nuclear and Radiological Sciences and Technology (INRASTES), National Centre for Scientific Research “Demokritos”, Aghia Paraskevi Attikis, Athens, Greece; Department of Medical Oncology, Papageorgiou Hospital, Aristotle University of Thessaloniki School of Medicine, Thessaloniki, Greece; Department of Medical Science, University of Turin, Turin, Italy; AO Città della Salute e della Scienza, Turin, Italy; Section of Medical Oncology, Department of Surgical and Oncological Sciences, University of Palermo, Palermo, Italy; Department of Oncology and Haematology, University of Modena and Reggio Emilia, Modena, Italy; Unit of Medical Genetics, Department of Biomedical, Experimental and Clinical Sciences, University of Florence, Florence, Italy; Unit of Hereditary Cancer, Department of Epidemiology, Prevention and Special Functions, IRCCS (Scientific Institute of Hospitalization and Care), AOU San Martino - IST National Institute for Cancer Research, Genoa, Italy; Molecular and Nutritional Epidemiology Unit, Cancer Research and Prevention Institute (ISPO), Florence, Italy; Unit of Genetic Counselling, Medical Oncology Department, Regina Elena National Cancer Institute, Rome, Italy; Unit of Molecular Bases of Genetic Risk and Genetic Testing, Department of Preventive and Predictive Medicine, IRCCS (Scientific Institute of Hospitalization and Care), National Cancer Institute (INT), 20133 Milan, Italy; Unit of Medical Genetics, Department of Preventive and Predictive Medicine, IRCCS (Scientific Institute of Hospitalization and Care), National Cancer Institute (INT), Milan, Italy; Division of Cancer Prevention and Genetics, European Institute of Oncology (IEO), Milan, Italy; Division of Experimental Oncology, CRO Aviano National Cancer Institute, Aviano, PN Italy; IFOM, FIRC (Italian Foundation for Cancer Research) Institute of Molecular Oncology, Milan, Italy; Cogentech Cancer Genetic Test Laboratory, Milan, Italy; National Cancer Institute “Giovanni Paolo II”, Bari, Italy; Clinical Cancer Genetics, City of Hope Clinical Cancer Genetics Community Research Network, Duarte, CA USA; Human Genetics Group, Human Cancer Genetics Program, Spanish National Cancer Centre (CNIO), Madrid, Spain; Biomedical Network on Rare Diseases (CIBERER), Madrid, Spain; Human Genetics Group, Spanish National Cancer Centre (CNIO), Madrid, Spain; Human Genotyping (CEGEN) Unit, Human Cancer Genetics Program, Spanish National Cancer Research Centre (CNIO), Madrid, Spain; Cancer Division, QIMR Berghofer Medical Research Institute, Brisbane, Australia; Department of Clinical Genetics, Rigshospitalet, Copenhagen University Hospital, Copenhagen, Denmark; Department of Oncology, Rigshospitalet, Copenhagen University Hospital, Copenhagen, Denmark; Center for Genomic Medicine, Rigshospitalet, Copenhagen University Hospital, Copenhagen, Denmark; Department of Medical Oncology, Beth Israel Deaconess Medical Center, Boston, MA USA; State Research Institute Centre for Innovative Medicine, Vilnius, Lithuania; Department of Dermatology, Huntsman Cancer Institute, University of Utah School of Medicine, Salt Lake City, UT USA; Department of Medicine, Huntsman Cancer Institute, University of Utah School of Medicine, Salt Lake City, UT USA; Department of Clinical Genetics, Fox Chase Cancer Center, Philadelphia, PA USA; Department of Pathology & Molecular Medicine, Juravinski Hospital and Cancer Centre, McMaster University, Hamilton, ON Canada; Department of Epidemiology, Mailman School of Public Health, Columbia University, New York, NY USA; Department of Epidemiology, Cancer Prevention Institute of California, Fremont, CA USA; Genetic Epidemiology Laboratory, Department of Pathology, University of Melbourne, Parkville, Australia

**Keywords:** Male breast cancer, *BRCA1/2*, Pathology, Histologic grade, Genotype–phenotype correlations

## Abstract

**Background:**

*BRCA1* and, more commonly, *BRCA2* mutations are associated with increased risk of male breast cancer (MBC). However, only a paucity of data exists on the pathology of breast cancers (BCs) in men with *BRCA1/2* mutations. Using the largest available dataset, we determined whether MBCs arising in *BRCA1/2* mutation carriers display specific pathologic features and whether these features differ from those of *BRCA1/2* female BCs (FBCs).

**Methods:**

We characterised the pathologic features of 419 *BRCA1/2* MBCs and, using logistic regression analysis, contrasted those with data from 9675 *BRCA1/2* FBCs and with population-based data from 6351 MBCs in the Surveillance, Epidemiology, and End Results (SEER) database.

**Results:**

Among *BRCA2* MBCs, grade significantly decreased with increasing age at diagnosis (*P* = 0.005). Compared with *BRCA2* FBCs, *BRCA2* MBCs were of significantly higher stage (*P* for trend = 2 × 10^−5^) and higher grade (*P* for trend = 0.005) and were more likely to be oestrogen receptor–positive [odds ratio (OR) 10.59; 95 % confidence interval (CI) 5.15–21.80] and progesterone receptor–positive (OR 5.04; 95 % CI 3.17–8.04). With the exception of grade, similar patterns of associations emerged when we compared *BRCA1* MBCs and FBCs. *BRCA2* MBCs also presented with higher grade than MBCs from the SEER database (*P* for trend = 4 × 10^−12^).

**Conclusions:**

On the basis of the largest series analysed to date, our results show that *BRCA1*/*2* MBCs display distinct pathologic characteristics compared with *BRCA1*/*2* FBCs, and we identified a specific *BRCA2-*associated MBC phenotype characterised by a variable suggesting greater biological aggressiveness (i.e., high histologic grade). These findings could lead to the development of gender-specific risk prediction models and guide clinical strategies appropriate for MBC management.

**Electronic supplementary material:**

The online version of this article (doi:10.1186/s13058-016-0671-y) contains supplementary material, which is available to authorized users.

## Background

Male breast cancer (MBC) is a rare disease. It accounts for less than 1 % of all breast cancers and less than 1 % of all cancers in men. The annual incidence is estimated at about 1 per 100,000 men worldwide [1], and lifetime risk is less than 1 in 1000. Incidence rates for MBC increase linearly and steadily with age, with the mean age at diagnosis being between 60 and 70 years [2]. Family history of breast cancer is an important risk factor for developing MBC, suggesting the importance of genetic factors in MBC susceptibility [3, 4]. Mutations in the two major high-penetrance breast cancer genes, *BRCA1* (breast cancer 1, early onset gene) and predominantly *BRCA2* (breast cancer 2, early onset gene), account for approximately 10 % of MBCs outside populations with *BRCA* founder mutations [5]. The lifetime risk of developing MBC has been estimated to be in the range of 1–5 % for *BRCA1* and 5–10 % for *BRCA2* mutation carriers, compared with a risk of 0.1 % in the general population [6–9].

MBC is recognised as being a hormone-dependent malignancy, and it is widely accepted as an oestrogen-driven disease, specifically related to hyperestrogenism [10]. In the general population, MBC is similar to late-onset, post-menopausal, oestrogen receptor–/progesterone receptor–positive (ER+/PR+) female breast cancer (FBC). However, compared with FBC, MBC has been reported to occur later in life, present at a higher stage and display lower histologic grade, with a higher proportion of ER+ and PR+ tumours [11].

There is increasing evidence suggesting that MBC may be a group of molecularly and clinically heterogeneous malignancies which differ from those seen in women [12]. It is well known that breast cancer in women is a heterogeneous disease. Breast cancers arising in female *BRCA1* mutation carriers display characteristic pathologic features, including distinct morphology (i.e., carcinomas with medullary features) and a triple-negative phenotype [i.e., ER−, PR−, human epidermal growth factor receptor 2–negative (HER2−)] in the majority. In contrast, *BRCA2* breast tumours are a more heterogeneous group, being broadly similar to non-*BRCA*–associated breast tumours, which more closely resemble post-menopausal FBCs, although with a tendency to be of high grade and HER2− [13].

Current knowledge of the pathologic characteristics of breast cancers arising in male *BRCA1/2* mutation carriers is limited, owing to the small number of carriers included in individual studies [14–17]. In a study including 50 male *BRCA1/2* mutation carriers, it was suggested that *BRCA2* MBCs may represent a subgroup of tumours with a peculiar phenotype not identified in FBC and characterised by an aggressive biological behaviour [16]. Furthermore, in a study including 28 male *BRCA1/2* mutation carriers, a possible *BRCA2* phenotype characterised by micropapillary histology was suggested [17]. In other, smaller studies, *BRCA2* MBCs were associated with younger age at diagnosis and positive lymph node status [14, 15].

In this study, we report pathology data characteristics of 419 *BRCA1/2* MBCs derived from the Consortium of Investigators of Modifiers of *BRCA1/2* (CIMBA), who conducted the largest study of its kind to date. The main objective of our study was to characterise the pathologic features of *BRCA1/2* MBCs and contrast those with the characteristics of *BRCA1/2* FBCs, as well as with MBCs in the general population.

## Methods

### CIMBA study participants

CIMBA collects data on male and female *BRCA1* or *BRCA2* pathogenic mutation carriers older than 18 years of age, with the majority recruited through cancer genetics clinics [18]. CIMBA data were submitted by 55 study groups in 24 countries based in Europe, North America and Australia. Pathology data from MBC cases for the present analysis were collected by 35 study groups (Additional file 1). Key variables collected for all CIMBA patients include year of birth, age at cancer diagnosis (breast, ovarian or prostate cancers), age at last observation, family membership, race and/or ethnicity and information on applicable prophylactic surgeries. This work was restricted to male and female mutation carriers who had been diagnosed with breast cancer and were of self-reported European ancestry. The number of male mutation carriers of non-European ancestry (2 *BRCA1* and 20 *BRCA2*) was too small to allow a meaningful analysis. These subjects were excluded from the analysis.

A signed informed consent form was obtained from study participants. All participating studies were approved by local ethical review committees (Additional file 2).

### Tumour pathology data

MBC pathology data were obtained from a range of sources, namely medical, pathology or tumour registry records and immunohistochemical staining and/or scoring of tissue microarrays (TMAs) (Additional file 3). The data included information on ER, PR and HER2 status; morphological subtype; lymph node involvement; TNM (tumour, node, metastasis) staging; and histologic grade. For ER, PR and HER2, status was classified as negative or positive. The vast majority of centres employed a cut-off of either ≥10 % or ≥1 % of tumour nuclei staining positive to define ER/PR receptor positivity, which was not centrally reclassified, owing to the low proportion of records with supporting staining data (Additional file 3). HER2 status was determined using immunohistochemistry (IHC) to detect strong complete membrane staining (with 3+ considered positive) with in situ hybridisation to detect HER2 gene amplification in equivocal cases. Consistency checks were performed to validate receptor data against supplementary scoring information when provided. Central pathology review was not performed.

Each carcinoma was assigned to a morphologic subgroup (ductal, lobular, medullary, other), which was confirmed using the World Health Organisation International Classification of Diseases 0 code for the classification of tumour type when present. Lymph node status, along with the number of nodes showing metastatic carcinoma, was provided when available. Staging data were based on the *AJCC Cancer Staging Manual, Sixth Edition* [19], with data provided on overall stage and its major attributes (primary tumour size, regional lymph node involvement and presence of distant metastasis). Histologic grade was determined by local pathologists using modifications of the Scarff-Bloom-Richardson histological grading system as grade 1, 2 or 3. Pathology data for FBCs included in the study are described in detail elsewhere [13].

### SEER data

We obtained MBC pathology data from the SEER 18 Registries Database for cases diagnosed from 1973 to 2011 [20]. For this study, we selected only male Caucasian cases diagnosed with invasive breast cancer. For SEER cases, pathology characteristics included age at diagnosis; morphologic subgroup; tumour grade; lymph node status; adjusted stage based on the *AJCC Cancer Staging Manual, Sixth Edition* [19]; ER, PR and HER2 status. Tumour grade was classified as grade 1 (well differentiated), grade 2 (moderately differentiated) or grade 3 (poorly differentiated).

SEER includes unselected MBCs, most of which are of unknown *BRCA1/2* mutation status. On the basis of published data [3, 21, 22], about 10 % of MBC cases are expected to be due to *BRCA1* or *BRCA2* mutations.

### Statistical methods

Logistic regression was used to assess the association between pathologic characteristics and male *BRCA1/2* mutation carrier status, as well as to compare pathologic characteristics with data from female *BRCA1/2* mutation carriers and from male breast tumours arising in the general population using SEER data. In the logistic regression analysis, each pathologic characteristic was treated as the explanatory variable. The outcome variables were *BRCA* mutation status (*BRCA1/BRCA2*), sex (female/male) and carrier status (general population/*BRCA1* mutation carrier and general population/*BRCA2* mutation carrier), with the first term used as the reference group. For assessment of continuous or ordered variables, such as age at diagnosis, stage and grade, tests for trend were also performed.

Analyses within CIMBA data were adjusted for age at diagnosis and country of origin, whereas comparisons between CIMBA and SEER data were adjusted only for age at diagnosis. In addition, an adjustment for calendar year of diagnosis was included in all analyses, based on the following groupings: up to 1990, 1991–2000 and after 2000. A robust variance approach was used to allow for dependencies between related individuals. All analyses were carried out using Stata v13 software (StataCorp, College Station, TX, USA).

## Results

### Pathologic characteristics of MBC in *BRCA1* and *BRCA2* mutation carriers

Information was available for 419 MBC cases, including 375 *BRCA2* and 44 *BRCA1* mutation carriers (Additional file 1). Median age at MBC diagnosis was 62 years [interquartile range (IQR) 16] for *BRCA2* mutation carriers and 62 years (IQR 18) for *BRCA1* mutation carriers.

The analysis was restricted to carriers diagnosed with invasive breast cancer (326 *BRCA2* and 40 *BRCA1*) (Additional file 4). The majority of tumours were invasive ductal carcinoma in both *BRCA2* (95.1 %) and *BRCA1* (100 %) carriers. Among tumours with data on stage and grade, the majority of *BRCA2* mutation carriers presented with stage 2 disease (47 %) and tumours of histologic grade 3 (56.7 %), whereas the majority of *BRCA1* mutation carriers presented with stage 3–4 disease (42.9 %) and histologic grade 3 tumours (69.2 %). Among tumours with ER, PR and HER2 data, 96.7 % were ER+, 86.8 % were PR+ and 83.4 % were HER2− in *BRCA2* mutation carriers, vs. 90.3 % ER+, 78.6 % PR+ and 89.5 % HER2− in *BRCA1* mutation carriers.

**Fig. 1:**
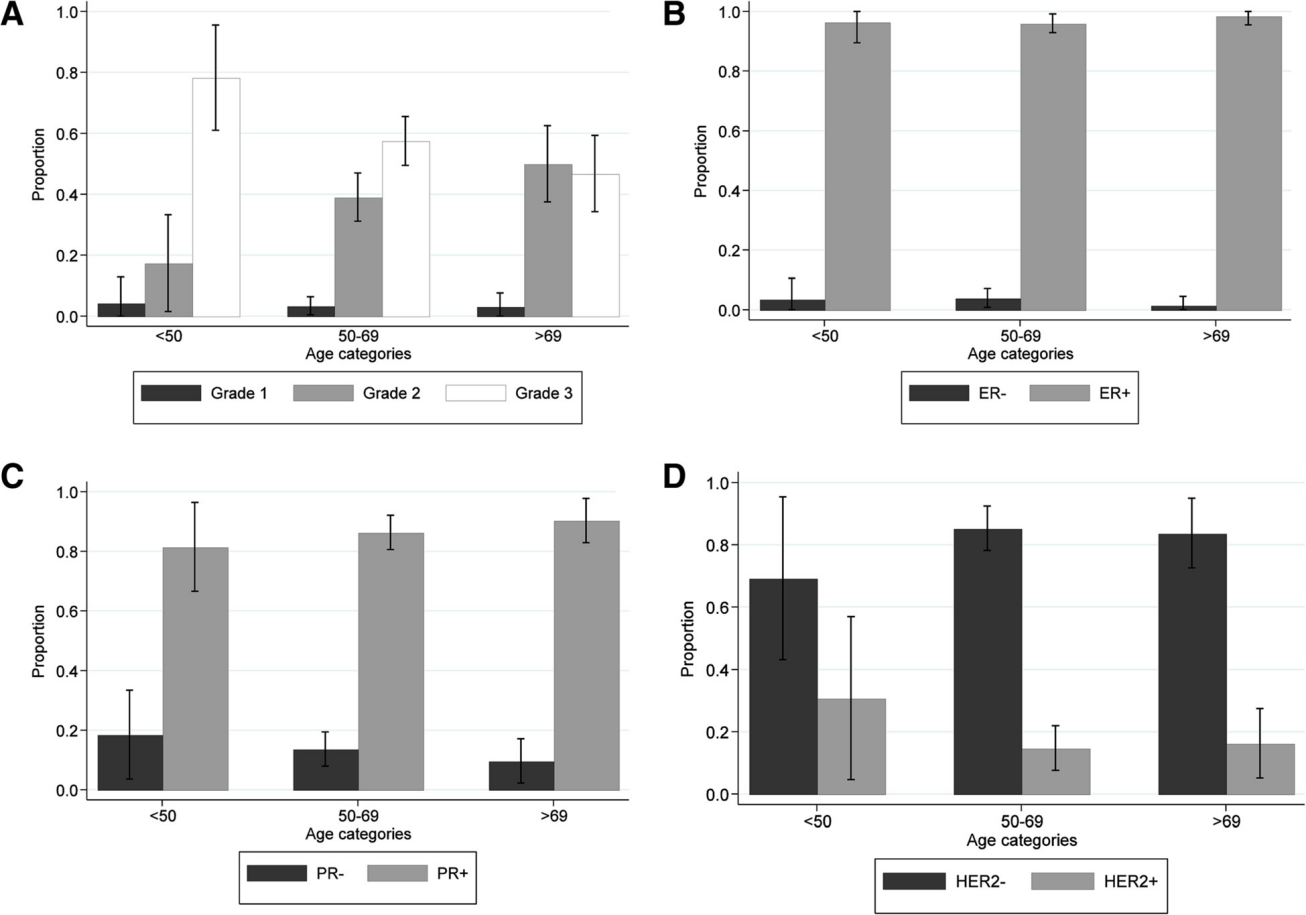
Age-specific proportion of *BRCA2* (breast cancer 2, early onset gene) male breast cancers according to pathologic characteristics. **a** Grade. **b** Oestrogen receptor (ER) status. **c** Progesterone receptor (PR) status. **d** Human epidermal growth factor receptor 2 (HER2) status. Error bars represent confidence intervals associated with each proportion

Age at diagnosis was inversely associated with grade in *BRCA2* mutation carriers (grade 1/2 vs. grade 3, *P* = 0.005), with no evidence for differences in ER, PR and HER2 distributions by age (test for differences *P* > 0.05 for all) (Fig. 1). Furthermore, there was no evidence of association between grade and ER or PR status (*P* values for trend = 0.50 and 0.78, respectively). For *BRCA1* mutation carriers, no differences in age-specific proportions of tumours by grade or ER, PR and HER2 status were observed, but their numbers were small (data not shown).

When we compared the pathologic characteristics of MBC in *BRCA1* and *BRCA2* mutation carriers, we observed no statistically significant differences. However, tumours in *BRCA1* mutation carriers were more likely to present with more advanced stage (42.9 % vs. 23.5 %, *P* for trend = 0.11) and were more frequently ER− (9.7 % vs. 3.3 %, *P* = 0.17) and PR− (21.4 % vs. 13.2 %, *P* = 0.27) than tumours in *BRCA2* mutation carriers (Additional file 4).

### Characterisation of *BRCA2* MBCs: comparison with *BRCA2* FBC and with MBC in the general population

We evaluated possible pathologic differences between invasive breast cancers arising in male and female *BRCA*2 mutation carriers by comparing available data from female mutation carriers with breast cancer in the CIMBA dataset. Data from 3750 country-matched female *BRCA2* mutation carriers diagnosed with invasive breast cancer were included in this analysis (Table 1). The results revealed that there were significantly fewer invasive lobular carcinomas among male *BRCA2* mutation carriers than among female *BRCA2* mutation carriers [odds ratio (OR) 0.14, 95 % confidence interval (CI) 0.05–0.43]. In addition, compared with *BRCA2* FBCs, *BRCA2* MBCs were of significantly higher stage (*P* for trend = 2.14 × 10^−5^) and higher grade (*P* for trend = 0.005), presented more frequently with lymph node involvement (OR 1.55, 95 % CI 1.12–2.14) and were more likely to be ER+ (OR 10.59, 95 % CI 5.15–21.80), PR+ (OR 5.04; 95 % CI 3.17–8.04) and non–triple-negative (OR 0.05, 95 % CI 0.01–0.22). Associations with stage and nodal, ER and PR status remained significant after adjustment for grade.

**Table 1:** Pathology of invasive *BRCA2* female and male breast tumours and ORs in predicting male *BRCA2* mutation carrier status

	Females		Males		Unadjusted OR (95 % CI)	Adjusted OR^a^ (95 % CI)
	Number	Percent	Number	Percent		
Total^b^	3750		326			
Morphology						
Ductal carcinoma	2693	83.6	253	95.1	Reference	Reference
Lobular carcinoma	276	8.6	4	1.5	**0.15 (0.06–0.41)**	**0.14 (0.05–0.43)**
Medullary carcinoma	60	1.9	2	0.8	0.35 (0.09–1.46)	0.46 (0.10–2.11)
Other	193	6.0	7	2.6	**0.39 (0.18–0.83)**	0.54 (0.24–1.23)
TNM stage						
0–1	560	40.2	44	29.5	Reference	Reference
2	629	45.1	70	47.0	1.42 (0.95–2.10)	**1.97 (1.20–3.23)**
3–4	205	14.7	35	23.5	**2.17 (1.37–3.44)**	**3.55 (1.96–6.44)**
Histologic grade						
Grade 1	149	5.9	8	3.5	Reference	Reference
Grade 2	1057	41.7	92	39.8	1.62 (0.77–3.41)	1.88 (0.76–4.67)
Grade 3	1329	52.4	131	56.7	1.84 (0.88–3.83	**2.66 (1.08–6.55)**
Lymph node status						
Negative	1398	52.4	123	50.2	Reference	Reference
Positive	1270	47.6	122	49.8	1.09 (0.84–1.43)	**1.55 (1.12–2.14)**
ER status						
Negative	650	22.7	8	3.3	Reference	Reference
Positive	2211	77.3	236	96.7	**8.67 (4.26–17.66)**	**10.59 (5.15–21.80)**
PR status						
Negative	892	35.0	30	13.2	Reference	Reference
Positive	1654	65.0	198	86.8	**3.56 (2.41–5.26)**	**5.04 (3.17–8.04)**
HER2 status						
Negative	1404	85.9	126	83.4	Reference	Reference
Positive	230	14.1	25	16.6	1.21 (0.77–1.90)	1.22 (0.70–2.11)
Subtypes						
ER+ and/or PR+, HER2−	1112	69.8	118	81.9	Reference	Reference
ER+ and/or PR+, HER2+	182	11.4	22	15.3	1.14 (0.70–1.84)	1.18 (0.65–2.13)
ER−, PR−, HER2+	40	2.5	2	1.4	0.47 (0.11–1.98)	0.42 (0.09–1.98)
Triple-negative (ER−, PR−, HER2−)	260	16.3	2	1.4	**0.07 (0.02–0.30)**	**0.05 (0.01–0.22)**
ER+ and/or PR+, HER2− vs. others					**0.51 (0.33–0.79)**	**0.42 (0.25–0.70)**

*BRCA2* breast cancer 2, early onset gene, *CI* confidence interval, *ER* oestrogen receptor, *HER2* human epidermal growth factor receptor 2, *OR* odds ratio, *PR* progesterone receptor, *TNM* tumour, node, metastasis

Significant results are indicated by boldface type

^a^Analyses adjusted for country, age at diagnosis and calendar year of diagnosis

^b^Some data for each pathologic feature are not available

We then compared pathologic features of MBC arising in *BRCA2* mutation carriers with characteristics of MBC in the general U.S. population as represented by SEER. We extracted pathology data of 6351 men with invasive breast cancer from the SEER 18 database. There were no statistically significant differences in pathology characteristics between MBCs arising in *BRCA2* mutation carriers and those arising in the general population, with the exception of grade and lymph node status (Table 2). Male *BRCA2* mutation carriers more frequently had grades 2 and 3 tumours than grade 1 tumours, as compared with MBC cases from the general population (grade 2 vs. grade 1 OR 2.98, 95 % CI 1.44–6.19; grade 3 vs. grade 1 OR 5.53, 95 % CI 2.69–11.39; *P* for trend = 4.52 × 10^−12^). Moreover, *BRCA2* mutation carriers presented more frequently with lymph node involvement than MBC cases from the general population, a difference that was not significant when adjusted for age at diagnosis and/or grade.

**Table 2:** Pathology of invasive MBCs in the general population from SEER and *BRCA2* MBCs and ORs in predicting male *BRCA2* mutation carrier status

	SEER		*BRCA2* carriers		Unadjusted OR (95 % CI)	Adjusted OR^a^ (95 % CI)
	Number	Percent	Number	Percent		
Total^b^	6351		326			
Morphology						
Ductal carcinoma	5265	86.2	253	95.1	Reference	Reference
Lobular carcinoma	82	1.5	4	1.5	1.02 (0.37–2.79)	1.00 (0.36–2.74)
Medullary Carcinoma	16	0.3	2	0.8	2.60 (0.59–11.38)	2.34 (0.52–10.39)
TNM stage						
0–1	1699	34.9	44	29.5	Reference	Reference
2	1990	40.9	70	47.0	1.36 (0.93.–1.99)	1.37 (0.93–2.01)
3–4	1181	24.2	35	23.5	1.14 (0.73–1.77)	1.11 (0.72–1.73)
Histologic grade						
Grade 1	632	12.9	8	3.5	Reference	Reference
Grade 2	2432	49.7	92	39.8	**2.99 (1.44–6.19)**	**2.98 (1.44–6.19)**
Grade 3	1834	37.4	131	56.7	**5.64 (2.75–11.60)**	**5.53 (2.69–11.39)**
Lymph node status						
Negative	2773	58.0	123	50.2	Reference	Reference
Positive	2009	42.0	122	49.8	**1.37 (1.05–1.78)**	1.28 (0.98–1.67)
ER status						
Negative	229	5.3	8	3.3	Reference	Reference
Positive	4064	94.7	236	96.7	1.66 (0.81–3.41)	1.95 (0.93–4.06)
PR status						
Negative	627	15.0	30	13.2	Reference	Reference
Positive	3562	85.0	198	86.8	1.16 (0.79–1.72)	1.30 (0.88–1.92)
HER2 status						
Negative	627	87.8	126	83.4	Reference	Reference
Positive	87	12.2	25	16.6	1.43 (0.88–2.32)	1.30 (0.79–2.13)
Subtypes						
ER+ and/or PR+, HER2−	608	87.5	118	81.9	Reference	Reference
ER+ and/or PR+, HER2+	80	11.5	22	15.3	1.42 (0.85–2.36)	1.28 (0.76–2.17)
ER−, PR−, HER2+	7	1.0	2	1.4	1.47 (0.30–7.18)	1.09 (0.22–5.45)
Triple-negative (ER−, PR−, HER2−)	0	0.0	2	1.4	–	–
ER+ and/or PR+, HER2− vs. others					1.54 (0.95–2.49)	1.38 (0.84–2.27)

*BRCA2* breast cancer 2, early onset gene, *CI* confidence interval, *ER* oestrogen receptor, *HER2* human epidermal growth factor receptor 2, *OR* odds ratio, *PR* progesterone receptor, *TNM* tumour, node, metastasis

Significant results are indicated by boldface type

^a^Analyses adjusted for age at diagnosis and calendar year of diagnosis

^b^Some data for each pathologic feature are not available

### Characterisation of *BRCA1* MBCs: comparison with *BRCA1* FBC and with MBC in the general population

A total of 5925 country-matched female *BRCA1* mutation carriers diagnosed with invasive breast cancer were compared with our *BRCA1* MBC series, which revealed that MBCs were of significantly higher stage (stage 3–4 vs. stage 1 OR 17.59, 95 % CI 3.47–89.03; *P* for trend = 0.001) and presented more frequently with lymph node involvement (OR 2.19, 95 % CI 1.03–4.65) than FBCs in *BRCA1* mutation carriers (Additional file 5). The association with stage remained significant after adjusting for ER and PR status. Moreover, *BRCA1* MBCs were more likely to be ER+ (OR 20.22, 95 % CI 5.91–69.17), PR+ (OR 13.76, 95 % CI 5.31–35.67) and non–triple-negative (OR 0.03, 95 % CI 0.00–0.25). The associations with ER and PR status remained significant after adjustment for stage. There was no statistically significant difference in the distribution of histologic grade among male and female *BRCA1* breast cancers.

The comparison between MBCs arising in *BRCA1* mutation carriers with those of 6351 MBCs from the SEER database showed no significant differences in pathologic characteristics (Additional file 6). However, *BRCA1* male breast tumours trended toward higher grade compared with those in the general population (*P* for trend = 0.003).

## Discussion

To date, most of the available knowledge on MBC is based on MBC arising in the general population, whose *BRCA1/2* mutation status is largely unknown. In this study, we sought to determine whether MBC arising in *BRCA1* and *BRCA2* mutation carriers displayed specific pathologic characteristics. We used data on 419 MBCs with *BRCA1* and *BRCA2* mutations from an international consortium (CIMBA). The CIMBA series represents the largest collection of MBCs arising in *BRCA1* and *BRCA2* mutation carriers to date. In our series, the majority of MBC cases (375 of 419, 89.5 %) were *BRCA2* mutation carriers, a finding which corroborates prior, smaller studies.

In this study, we conducted the first comparison of the pathologic features of breast cancer arising in male and female *BRCA1/2* mutation carriers, taking advantage of the previously collected pathology data from female *BRCA1/2* mutation carriers assembled by CIMBA [13].

We found that breast cancer in male *BRCA2* mutation carriers was of significantly higher stage and histologic grade, and was more frequently ER+ and PR+, than breast cancer in female *BRCA2* mutation carriers. Advanced stage disease at breast cancer diagnosis is more frequently observed in men than in women [23]. In general, this is thought to reflect diagnostic delay in a population unaware of its risk and (appropriately) not encouraged to undergo routine breast cancer screening. Furthermore, although breast cancer primaries in men tend to be slightly smaller than those in women when they are first diagnosed, they more often have locoregional metastasis at presentation. Indeed, we found that male *BRCA2* mutation carriers presented more frequently with lymph node involvement than breast cancer in female mutation carriers.

It is known that MBC presents with lower histologic grade tumours than FBC in the general population [11]. In contrast, in the present study, we showed that MBC associated with *BRCA2* mutations presents with higher histologic grade than both breast cancer in female *BRCA2* mutation carriers and MBC in the general population from SEER.

We observed that the majority of *BRCA2* MBCs are of grades 2 and 3. However, grade 3 tumours were more frequent among male *BRCA2* mutation carriers diagnosed at younger ages (younger than age 50 years) than among those diagnosed at older ages, whereas grade 2 tumours showed an inverse trend. Age-specific proportions of MBCs stratified by grade show that grade 3 significantly decreased with increasing age in male *BRCA2* mutation carriers. These results may indicate that young male *BRCA2* mutation carriers could be susceptible to more aggressive (i.e., high-grade) breast cancer. Differences in grade among male breast carcinomas by age may be an indicator of a biologic complexity in MBC, as suggested in FBC [24].

In a previous, single-country case series, MBCs associated with *BRCA2* mutations were found to be of higher grade than non-*BRCA2* MBC [16]. In the present study, we confirmed this association in a large, multicentre series and showed that this association was age-specific. The identification of a specific *BRCA2*-associated phenotype suggestive of an aggressive behaviour might define a subset of MBC patients (i.e., patients with high-grade breast tumours and with young age at diagnosis) who may particularly benefit from adjuvant chemotherapy [2, 25].

We also showed that high-grade breast tumours were more likely to arise in male than in female *BRCA2* mutation carriers, indicating that *BRCA2* mutations might be associated with different breast cancer phenotypes in men and in women. It has been suggested that high grade is a surrogate for proliferation, and, although the evidence is conflicting, this may add to the understanding of the molecular differences of MBC and FBC.

MBC is recognised as being primarily a hormone-dependent malignancy, and, in general, MBC is described as being more frequently ER+ and PR+ than FBC [10, 11, 23]. In the present study, we showed that *BRCA2* MBCs are more likely than *BRCA2* FBCs to be ER+ and PR+, thus suggesting that susceptibility to hereditary breast cancer may be influenced by differences in hormonal background between male and female *BRCA2* mutation carriers.

Invasive lobular carcinomas are very rare in men, accounting for only about 2 % of all MBCs [23, 26]. We also found significantly fewer lobular carcinomas among male than female *BRCA2* mutation carriers. However, it is worth noting that breast cancers in female *BRCA2* mutation carriers frequently show a lobular morphology [13], thus suggesting differences in the pathogenic mechanisms of male and female *BRCA2* breast cancer.

The number of MBC cases with *BRCA1* mutations in our datasets was much smaller than the number of *BRCA2* mutation carriers, and our results in this subset of patients should therefore be interpreted with caution. We found that *BRCA1* MBC cases were of significantly higher stage, and more frequently ER+ and PR+, than *BRCA1* FBCs. Despite the small sample size, our results suggest that hormone receptor pathways also are a driving force in *BRCA1* MBC. It is well known that most of the breast tumours arising in female *BRCA1* mutation carriers tend to be ER− and PR−, with a small percentage being ER+ [13, 27, 28]. Given that both ER− and ER+ *BRCA1* breast cancers seem to originate from a common luminal progenitor cell population, it has been suggested that ER status of breast cancer occurring in *BRCA1* mutation carriers may be under control of different molecular mechanisms [29]. The finding that MBCs associated with *BRCA1* mutations are frequently ER+ suggests that the hormonal milieu may be a mechanism controlling ER status in *BRCA1* tumours. The different hormonal background between males and females and the absence of hormone exposures related to reproductive history in males as compared with FBC may also influence biologic and molecular mechanisms underlying the pathologic differences between MBC and FBC. Following the findings in the present study, future studies are warranted which focus on the comprehensive somatic and molecular profiling of MBC and FBC in mutation carriers. Such studies could provide new insights into the complex nature of the origin and evolution of MBC and FBC.

Interestingly, we found no statistically significant differences in the pathologic characteristics between MBCs in *BRCA1*/*2* mutation carriers and those in the general population, with the exception of histologic grade. Male *BRCA2* mutation carriers more frequently have grade 2/3 vs. grade 1 tumours, compared with the large, unselected population of MBC cases from SEER. A similar trend also was observed for *BRCA1* mutation carriers. These findings suggest that, although MBCs arising in male *BRCA1*/*2* mutation carriers seem to be very similar to MBCs arising in the general population, according to morphologic and immunophenotypic features, they represent a subgroup characterised by aggressive biology.

The importance of histologic grade as a prognostic factor in breast cancer has been ascertained in FBC [30]. Recent data indicate that high-grade tumours are associated with shorter disease-free survival and overall survival rates in MBC patients [25]. Thus, on the basis of our results, we can suggest that *BRCA2* MBC may display an aggressive phenotype and possibly a more unfavourable prognosis. This is a question in need of additional survival data that we are planning to collect within CIMBA.

In this study, tumour pathology data were collected through several mechanisms, including medical records, pathology reports and TMAs. Given the global distribution of CIMBA study sites, central pathology review was not feasible. Laboratory methods for tissue preparation, IHC, biochemical assays, scoring systems and data interpretation vary widely (Additional file 3), and misclassifications cannot be excluded. Unfortunately, details of hormone receptor scoring for all mutation carriers were not available to standardise definitions across centres. However, data collected by CIMBA are more representative of typical assessment of pathology conducted in routine practice, and the distributions of hormone receptors’ status across different study centres and countries in CIMBA were generally consistent. There was some variation in the distribution of some variables, including ER status, probably due to changing assay thresholds and detection methods over time and from country to country. Therefore, adjustments based on calendar year of diagnosis and country of origin were included for all analyses. Missing data for some variables, including HER2 status, and the very small number of male *BRCA1* mutation carriers in the study may have impacted the statistical power to detect associations.

CIMBA collects data only on *BRCA1* and *BRCA2* mutation carriers. Therefore, to compare the tumour characteristics of MBC from the general population, we took advantage of the publicly available SEER data [20]. Although the U.S. SEER program is the largest source of epidemiologic information on the incidence and survival rates of cancer, it includes data from a single country, and this represents a limitation when attempting to generalise our findings to what one would expect in a collaborative international consortium. However, results from this study, based on a large, multicentre series, replicated previous findings of much smaller studies carried out in single populations [14–17], providing some reassurance that our results were not biased by the different selection of cases in SEER and in CIMBA. In addition, SEER includes MBCs that were not screened for *BRCA1* and *BRCA2* mutations, and it can be expected that about 10 % of those cases [3, 21, 22] may be due to *BRCA1* or *BRCA2* mutations. In future studies, researchers should aim to compare *BRCA1/2* MBC cases with those known not to have *BRCA1/2* mutations.

## Conclusions

Analysing the largest series of *BRCA1* and *BRCA2* breast cancers collected to date from both sexes, we have demonstrated that breast tumours arising in *BRCA1* and *BRCA2* mutation carriers display pathologic differences between males and females. Thus, our results add to the accumulating evidence that breast cancer may not be the same disease in both genders [12] and suggest that the heritable influence on breast cancer susceptibility may be context-dependent, perhaps influenced by the microenvironment (i.e., a different hormonal milieu in males and females).

Moreover, we identified a specific *BRCA2*-associated MBC phenotype characterised by higher histologic grade compared with both *BRCA2* FBC and MBC from a general population. This raises the possibility that *BRCA2* MBC may be more aggressive than its sporadic counterpart.

Overall, our findings could lead to the eventual development of clinical strategies appropriate for MBC management, and of gender-specific risk prediction models that might guide more targeted screening and surveillance programs for male mutation carriers.

## Additional files

Additional file 1:
**Male** ***BRCA1*** **and** ***BRCA2*** **mutation carriers by study group/country.** (DOCX 21 kb) Additional file 2:
**List of local ethics committees that granted approval for the access and use of the data in present study.** (DOCX 23 kb) Additional file 3:
**Methods and thresholds used to define the final marker variables for study groups providing MBC cases.** (DOCX 20 kb) Additional file 4:
**Pathology of** ***BRCA1*** **and** ***BRCA2*** **MBCs and ORs in predicting** ***BRCA2*** **mutation carrier status.** (DOCX 20 kb) Additional file 5:
**Pathology of invasive** ***BRCA1*** **female and male breast tumours and ORs in predicting male** ***BRCA1*** **mutation carrier status.** (DOCX 19 kb) Additional file 6:
**Pathology of invasive MBCs in the general population from SEER and** ***BRCA1*** **MBCs and ORs in predicting male** ***BRCA1*** **mutation carrier status.** (DOCX 19 kb)

## Abbreviations

BC: breast cancer
*BRCA1*: breast cancer 1, early onset gene
*BRCA2*: breast cancer 2, early onset gene
CI: confidence interval
CIMBA: Consortium of Investigators of Modifiers of *BRCA1/2*
ER: oestrogen receptor
FBC: female breast cancer
HER2: human epidermal growth factor receptor 2
IHC: immunohistochemistry
IQR: interquartile range
MBC: male breast cancer
OR: odds ratio
PR: progesterone receptor
SEER: Surveillance, Epidemiology, and End Results
TMA: tissue microarray
TNM: tumour, node, metastasis
