# Supplementary material for: Male breast cancer in BRCA1 and BRCA2 mutation carriers: pathology data from the Consortium of Investigators of Modifiers of BRCA1/2
Source: Breast Cancer Res. 2016 Feb 9;18:15. doi: 10.1186/s13058-016-0671-y (PMC4746828; doi:10.1186/s13058-016-0671-y)
Supplement: Additional file 1: — Male BRCA1 and BRCA2 mutation carriers by study group/country. (DOCX 21 kb) [file 13058_2016_671_MOESM1_ESM.docx]

**Additional file 1:** Male *BRCA1* and *BRCA2* mutation carriers by study group/country.

| **Study/Group** | **Country** | ***BRCA1* mutation carriers** | ***BRCA2* mutation carriers** | **Total** |
| --- | --- | --- | --- | --- |
| BCFR-ON | Canada | 1 | 5 | 6 |
| BCFR-UT | USA | 1 | 2 | 3 |
| BRICOH | USA | 1 | 19 | 20 |
| CBCS | Denmark | 0 | 4 | 4 |
| CNIO | Spain | 0 | 15 | 15 |
| CONSIT TEAM | Italy | 5 | 49 | 54 |
| CZ-BRCA | Czech Republic | 4 | 16 | 20 |
| DEMOKRITOS | Greece | 0 | 1 | 1 |
| EMBRACE | UK | 4 | 28 | 32 |
| FCCC | USA | 0 | 3 | 3 |
| G-FAST | Belgium | 0 | 10 | 10 |
| GC-HBOC | Germany | 10 | 55 | 65 |
| GEMO | France | 0 | 11 | 11 |
| HCSC | Spain | 0 | 6 | 6 |
| HEBCS | Finland | 0 | 1 | 1 |
| HEBON | Netherlands | 0 | 3 | 3 |
| HUNBOCS | Hungary | 2 | 16 | 18 |
| HVH | Spain | 0 | 7 | 7 |
| ICO | Spain | 1 | 8 | 9 |
| ILUH | Iceland | 0 | 12 | 12 |
| IOVHBOCS | Italy | 0 | 5 | 5 |
| IPOBCS | Portugal | 0 | 2 | 2 |
| KCONFAB | Australia | 5 | 26 | 31 |
| MAYO | USA | 1 | 3 | 4 |
| MSKCC | USA | 1 | 10 | 11 |
| MUV | Austria | 0 | 6 | 6 |
| NCI | USA | 1 | 1 | 2 |
| OCGN | Canada | 0 | 7 | 7 |
| OSU CCG | USA | 0 | 3 | 3 |
| OUH | Denmark | 2 | 10 | 12 |
| PBCS | Italy | 0 | 3 | 3 |
| UCHICAGO | USA | 0 | 4 | 4 |
| UPENN | USA | 4 | 14 | 18 |
| UPITT | USA | 0 | 7 | 7 |
| VFCTG | Australia | 1 | 3 | 4 |
| **Total** |  | **44** | **375** | **419** |
