# Supplementary material for: Male breast cancer in BRCA1 and BRCA2 mutation carriers: pathology data from the Consortium of Investigators of Modifiers of BRCA1/2
Source: Breast Cancer Res. 2016 Feb 9;18:15. doi: 10.1186/s13058-016-0671-y (PMC4746828; doi:10.1186/s13058-016-0671-y)
Supplement: Additional file 3: — Methods and thresholds used to define the final marker variables for study groups providing MBC cases. (DOCX 20 kb) [file 13058_2016_671_MOESM3_ESM.docx]

**Additional file 3:** Methods and thresholds used to define the final marker variables for study groups providing MBC cases.

| **Study** | **Marker** | **Source** | **Definition of positive status** |
| --- | --- | --- | --- |
|  |  |  |  |
| BCFR / OCGN | ER / PR | PRep, MRec, PRev, TReg | ≥1% stained nuclei |
| BRICOH | ER / PR | PRep, MRec, TReg | >15 fmoles/mg Cytosolic protein / As assigned by clinicians |
| CBCS | ER / PR | PRep | ≥10% stained nuclei |
| CNIO | ER / PR | MRec, TMAs | ≥10% stained nuclei or Allred score >2 |
| CONSIT TEAM | ER / PR | PRep, MRec | ≥10% stained nuclei or Allred score >2 or H-score ≥50 or >10 fmoles/mg Cytosolic protein |
| CZ-BRCA | ER / PR | PRep, MRec | None supplied |
| DEMOKRITOS | ER / PR | PRep, MRec, PRev | ≥10% stained nuclei or Allred score >2 |
| EMBRACE | ER / PR | PRep, MRec, TReg | ≥10% stained nuclei or Allred score >2 or H-score ≥50 |
| FCCC | ER / PR | PRev, TReg | None supplied |
| G-FAST | ER / PR | PRep | None supplied |
| GC-HBOC | ER / PR | MRec, PRev | ≥10% stained nuclei or Remmele score ≥1 |
| GEMO | ER / PR | MRec | ≥1% stained nuclei |
| HCSC | ER / PR | PRep, MRec, PRev | ≥10% stained nuclei or Allred score >2 |
| HEBCS | ER / PR | PRep, MRec, PRev, TMAs | ≥10% stained nuclei |
| HEBON | ER / PR | PRep | None supplied |
| HUNBOCS | ER / PR | PRep, MRec | None supplied |
| HVH | ER / PR | PRep, MRec | None supplied |
| ICO | ER / PR | PRep, PRev | ≥10% stained nuclei |
| ILUH | ER | PRep | ≥8 fmoles/mg Cytosolic protein |
| ILUH | PR | PRep | ≥25 fmoles/mg Cytosolic protein |
| IOVHBOCS | ER / PR | PRep, MRec | As assigned by clinicians |
| IPOBCS | ER / PR | MRec | ≥1% stained nuclei |
| KCONFAB | ER / PR | PRep, MRec, TReg, TMAs | Allred score >2 |
| MAYO | ER / PR | MRec | None supplied |
| MSKCC | ER / PR | PRep, MRec, TReg | None supplied |
| MUV | ER / PR | PRep, MRec, PRev | None supplied |
| NCI | ER / PR |  | None supplied |
| OSU CCG | ER / PR | PRep, MRec | None supplied |
| OUH | ER / PR | PRep, TReg | ≥1% stained nuclei |
| PBCS | ER / PR | PRep, MRec | ≥1% stained nuclei |
| UCHICAGO | ER / PR | MRec | ≥10% stained nuclei |
| UPENN | ER / PR | PRep, MRec | None supplied |
| UPITT | ER / PR | PRep, MRec | ≥1% stained nuclei |
| VFCTG | ER / PR | PRep | ≥1% stained nuclei or ≥10 fmoles/mg Cytosolic protein |

Source abbreviations:

PRep: Pathology Reports; MRec: Medical Records; PRev: Pathology Review; TReg: Tumour Registry; TMAs: Tissue Microarray

Composite scoring methods:

Allred score 0-8 (percent staining score 0-5 + intensity of staining 0-3); H-Score 0-300 (3 x percentage of strongly staining nuclei + 2 x percentage of moderately staining nuclei + percentage of weakly staining nuclei, giving a range of 0 to 300); Remmele score 0-12 (percent staining score 0-4 * intensity of staining 0-3).
