# Supplementary material for: Male breast cancer in BRCA1 and BRCA2 mutation carriers: pathology data from the Consortium of Investigators of Modifiers of BRCA1/2
Source: Breast Cancer Res. 2016 Feb 9;18:15. doi: 10.1186/s13058-016-0671-y (PMC4746828; doi:10.1186/s13058-016-0671-y)
Supplement: Additional file 4: — Pathology of BRCA1 and BRCA2 MBCs and ORs in predicting BRCA2 mutation carrier status. (DOCX 20 kb) [file 13058_2016_671_MOESM4_ESM.docx]

**Additional file 4:** Pathology of *BRCA1* and *BRCA2* MBCs and ORs in predicting *BRCA2* mutation carrier status.

|  | ***BRCA1*** |  | ***BRCA2*** |  | **Unadjusted OR (95%CI)** | **Adjusted OR^a^(95%CI)** |
| --- | --- | --- | --- | --- | --- | --- |
|  | ***N*** | ***%*** | ***N*** | ***%*** |  |  |
| **Total^b^** | 44 |  | 375 |  |  |  |
|  |  |  |  |  |  |  |
| **Behavior** |  |  |  |  |  |  |
| Invasive | 40 | 98% | 326 | 95% | - | - |
| in situ | 1 | 2% | 16 | 5% | - | - |
| **Morphology^c^** |  |  |  |  |  |  |
| Ductal carcinoma | 34 | 100.0 | 253 | 95.1 | - | - |
| Lobular carcinoma | 0 | 0.0 | 4 | 1.5 | - | - |
| Medullary carcinoma | 0 | 0.0 | 2 | 0.8 | - | - |
| Other | 0 | 0.0 | 7 | 2.6 | - | - |
| **TNM Stage^c^** |  |  |  |  |  |  |
| 0-1 | 2 | 14.3 | 44 | 29.5 | ref | ref |
| 2 | 6 | 42.9 | 70 | 47.0 | 0.53 (0.10-2.76) | 0.52 (0.11-2.57) |
| 3-4 | 6 | 42.9 | 35 | 23.5 | 0.27 (0.05-1.40) | 0.27 (0.05-1.43) |
| **Histologic grade^c^** |  |  |  |  |  |  |
| Grade 1 | 1 | 3.8 | 8 | 3.5 | ref | ref |
| Grade 2 | 7 | 26.9 | 92 | 39.8 | 1.64 (0.18-15.14) | 1.36 (0.18-10.07) |
| Grade 3 | 18 | 69.2 | 131 | 56.7 | 0.91 (0.11-7.74) | 0.68 (0.10-4.44) |
| **Lymph node status^c^** |  |  |  |  |  |  |
| Negative | 14 | 46.7 | 123 | 50.2 | ref | ref |
| Positive | 16 | 53.3 | 122 | 49.8 | 0.87 (0.40-1.86) | 0.91 (0.42-1.98) |
| **ER status^c^** |  |  |  |  |  |  |
| Negative | 3 | 9.7 | 8 | 3.3 | ref | ref |
| Positive | 28 | 90.3 | 236 | 96.7 | 3.16 (0.79-12.65) | 2.78 (0.65-12.04) |
| **PR status^c^** |  |  |  |  |  |  |
| Negative | 6 | 21.4 | 30 | 13.2 | ref | ref |
| Positive | 22 | 78.6 | 198 | 86.8 | 1.80 (0.67-4.81) | 1.81 (0.63-5.24) |
| **HER2 status^c^** |  |  |  |  |  |  |
| Negative | 17 | 89.5 | 126 | 83.4 | ref | ref |
| Positive | 2 | 10.5 | 25 | 16.6 | 1.69 (0.36-7.80) | 1.74 (0.29-10.55) |
| **Subtypes^c^** |  |  |  |  |  |  |
| ER and/or PR+, HER2- | 16 | 84.2 | 118 | 81.9 | ref | ref |
| ER and/or PR+, HER2+ | 1 | 5.3 | 22 | 15.3 | 2.98 (0.37-23.81) | 2.59 (0.27-25.00) |
| ER-, PR-, HER2+ | 1 | 5.3 | 2 | 1.4 | 0.27 (0.02-3.19) | 0.40 (0.02-7.94) |
| Triple Negative (ER-, PR-, HER2-) | 1 | 5.3 | 2 | 1.4 | 0.27 (0.02-3.19) | 0.19 (0.02-1.74) |

^a^ Analyses adjusted for country, age at diagnosis, and calendar year of diagnosis.

^b^ Some data for each pathologic feature are not available.

^c^Analyses restricted to invasive MBCs.
