# Supplementary material for: Male breast cancer in BRCA1 and BRCA2 mutation carriers: pathology data from the Consortium of Investigators of Modifiers of BRCA1/2
Source: Breast Cancer Res. 2016 Feb 9;18:15. doi: 10.1186/s13058-016-0671-y (PMC4746828; doi:10.1186/s13058-016-0671-y)
Supplement: Additional file 5: — Pathology of invasive BRCA1 female and male breast tumours and ORs in predicting male BRCA1 mutation carrier status. (DOCX 19 kb) [file 13058_2016_671_MOESM5_ESM.docx]

**Additional file 5:** Pathology of invasive *BRCA1* female and male breast tumors and ORs in predicting male *BRCA1* mutation carrier status.

|  | **Females** |  | **Males** |  | **Unadjusted OR (95%CI)** | **Adjusted OR^a^(95%CI)** |
| --- | --- | --- | --- | --- | --- | --- |
|  | ***N*** | ***%*** | ***N*** | ***%*** |  |  |
| **Total^b^** | 5,925 |  | 40 |  |  |  |
| **Morphology** |  |  |  |  |  |  |
| Ductal carcinoma | 4,164 | 82.4 | 34 | 100.0 | - | - |
| Lobular carcinoma | 122 | 2.4 | 0 | 0.0 | - | - |
| Medullary carcinoma | 407 | 8.1 | 0 | 0.0 | - | - |
| Other | 360 | 7.1 | 0 | 0.0 | - | - |
| **TNM Stage** |  |  |  |  |  |  |
| 0-1 | 1,074 | 21.3 | 2 | 5.9 | ref | ref |
| 2 | 1,085 | 21.5 | 6 | 17.6 | 2.97 (0.60-14.76) | 4.04 (0.86-18.91) |
| 3-4 | 192 | 3.8 | 6 | 17.6 | **16.78 (3.36-83.83)** | **17.59 (3.47-89.03)** |
| **Histologic grade** |  |  |  |  |  |  |
| Grade 1 | 102 | 2.6 | 1 | 3.8 | ref |  |
| Grade 2 | 752 | 19.1 | 7 | 26.9 | 0.95 (0.12-7.70) | 1.70 (0.32-8.95) |
| Grade 3 | 3,077 | 78.3 | 18 | 69.2 | 0.60 (0.08-4.43) | 2.47 (0.54-11.20) |
| **Lymph node status** |  |  |  |  |  |  |
| Negative | 2,733 | 65.0 | 14 | 46.7 | ref | ref |
| Positive | 1,472 | 35.0 | 16 | 53.3 | **2.12 (1.03-4.35)** | **2.19 (1.03-4.65)** |
| **ER status** |  |  |  |  |  |  |
| Negative | 3,340 | 76.0 | 3 | 9.7 | ref | ref |
| Positive | 1,053 | 24.0 | 28 | 90.3 | **29.60 (8.96-97.78)** | **20.22 (5.91-69.17)** |
| **PR status** |  |  |  |  |  |  |
| Negative | 3,185 | 79.3 | 6 | 21.4 | ref | ref |
| Positive | 829 | 20.7 | 22 | 78.6 | **14.09 (5.69-34.90)** | **13.76 (5.31-35.67)** |
| **HER2 status** |  |  |  |  |  |  |
| Negative | 2,410 | 89.9 | 17 | 89.5 | ref | ref |
| Positive | 272 | 10.1 | 2 | 10.5 | 1.04 (0.24-4.54) | 1.17 (0.22-6.24) |
| **Subtypes** |  |  |  |  |  |  |
| ER and/or PR+, HER2- | 592 | 23.0 | 16 | 84.2 | ref | ref |
| ER and/or PR+, HER2+ | 113 | 4.4 | 1 | 5.3 | 0.33 (0.04-2.50) | 0.43 (0.04-4.97) |
| ER-, PR-, HER2+ | 140 | 5.4 | 1 | 5.3 | 0.26 (0.03-2.02) | 0.39 (0.05-2.96) |
| Triple Negative (ER-, PR-, HER2-) | 1,731 | 67.2 | 1 | 5.3 | **0.06 (0.01-0.19)** | **0.03 (0.00-0.25)** |
| ER and/or PR+, HER2- vs Others |  |  |  |  | **0.06 (0.02-0.19)** | **0.08 (0.02-0.31)** |

^a^ Analyses adjusted for country, age at diagnosis, and calendar year of diagnosis.

^b^ Some data for each pathologic feature are not available.
