# Supplementary material for: Male breast cancer in BRCA1 and BRCA2 mutation carriers: pathology data from the Consortium of Investigators of Modifiers of BRCA1/2
Source: Breast Cancer Res. 2016 Feb 9;18:15. doi: 10.1186/s13058-016-0671-y (PMC4746828; doi:10.1186/s13058-016-0671-y)
Supplement: Additional file 6: — Pathology of invasive MBCs in the general population from SEER and BRCA1 MBCs and ORs in predicting male BRCA1 mutation carrier status. (DOCX 19 kb) [file 13058_2016_671_MOESM6_ESM.docx]

**Additional file 6:** Pathology of invasive MBCs in the general population from SEER and *BRCA1*  MBCs and ORs in predicting male *BRCA1* mutation carrier status.

|  | **SEER** |  | ***BRCA1*** |  | **Unadjusted OR (95%CI)** | **Adjusted OR^a^ (95%CI)** |
| --- | --- | --- | --- | --- | --- | --- |
|  | ***N*** | ***%*** | ***N*** | ***%*** |  |  |
| **Total^b^** | 6,351 |  | 40 |  |  |  |
| **Morphology** |  |  |  |  |  |  |
| Ductal carcinoma | 5,265 | 86.2 | 34 | 100.0 | - | - |
| Lobular carcinoma | 82 | 1.5 | 0 | 0.0 | - | - |
| Medullary Carcinoma | 16 | 0.3 | 0 | 0.0 | - | - |
| **TNM Stage** |  |  |  |  |  |  |
| 0-1 | 1,699 | 34.9 | 2 | 14.3 | Ref | ref |
| 2 | 1,990 | 40.9 | 6 | 42.9 | 2.56 (0.52-12.71) | 2.58 (0.52-12.82) |
| 3-4 | 1,181 | 24.2 | 6 | 42.9 | 4.32 (0.87-21.42) | 4.24 (0.86-20.96) |
| **Histologic grade** |  |  |  |  |  |  |
| Grade 1 | 632 | 12.9 | 1 | 3.8 | Ref | ref |
| Grade 2 | 2,432 | 49.7 | 7 | 26.9 | 1.82 (0.22-14.82) | 1.82 (0.22-14.86) |
| Grade 3 | 1,834 | 37.4 | 18 | 69.2 | 6.20 (0.83-46.57) | 6.25 (0.84-46.40) |
| **Lymph node status** |  |  |  |  |  |  |
| Negative | 2,773 | 58.0 | 14 | 46.7 | Ref | ref |
| Positive | 2,009 | 42.0 | 16 | 53.3 | 1.58 (0.77-3.24) | 1.48 (0.71-3.10) |
| **ER status** |  |  |  |  |  |  |
| Negative | 229 | 5.3 | 3 | 9.7 | Ref | ref |
| Positive | 4,064 | 94.7 | 28 | 90.3 | 0.53 (0.16-1.74) | 0.61 (0.19-2.06) |
| **PR status** |  |  |  |  |  |  |
| Negative | 627 | 15.0 | 6 | 21.4 | Ref | ref |
| Positive | 3,562 | 85.0 | 22 | 78.6 | 0.65 (0.26-1.60) | 0.75 (0.30-1.92) |
| **HER2 status** |  |  |  |  |  |  |
| Negative | 627 | 87.8% | 17 | 89.5 | Ref | ref |
| Positive | 87 | 12.2% | 2 | 10.5 | 0.85 (0.19-3.73) | 0.76 (0.17-3.41) |
| **Subtypes** |  |  |  |  |  |  |
| ER and/or PR+, HER2- | 608 | 87.5% | 16 | 84.2 | Ref | ref |
| ER and/or PR+, HER2+ | 80 | 11.5% | 1 | 5.3 | 0.47 (0.06-3.64) | 0.43 (0.06-3.28) |
| ER-, PR-, HER2+ | 7 | 1.0% | 1 | 5.3 | 5.43 (0.63-46.83) | 4.40 (0.45-42.92) |
| Triple Negative (ER-, PR-, HER2-) | 0 | 0.0% | 1 | 5.3 | - | - |
| ER and/or PR+, HER2- vs Others |  |  |  |  | 1.31 (0.37-4.59) | 1.16 (0.33-4.10) |

^a^ Analyses adjusted for age at diagnosis and calendar year of diagnosis.

^b^ Some data for each pathologic feature are not available.
